# Hemoglobin SC Sickle Cell Disease Presenting as Acute Ischemic Stroke With Moyamoya Syndrome in an Adult: A Case Report

**DOI:** 10.7759/cureus.93120

**Published:** 2025-09-24

**Authors:** Tejashree Nidiginti, Basil Afzal, Reshmi Roy, Farhan Baig, Balwant Rai

**Affiliations:** 1 General Internal Medicine, Calderdale and Huddersfield NHS Foundation Trust, Halifax, GBR; 2 Radiology, Calderdale and Huddersfield NHS Foundation Trust, Huddersfield, GBR

**Keywords:** cerebrovascular diseases, magnetic resonance angiography (mra), moyamoya disease and syndrome, neuroimaging findings, sickle cell disease (scd), stroke, transient ischemic attacks

## Abstract

Moyamoya disease is a rare condition where blood vessels at the base of the brain become narrow or blocked. This leads to the growth of small, fragile vessels that look like a “puff of smoke” on imaging tests like CT or MRI scans. These vascular changes predominantly involve the terminal portion of the internal carotid arteries and the proximal segments of the anterior and middle cerebral arteries, leading to progressive stenosis and the development of collateral networks. Although the exact cause of moyamoya disease is unknown, it increases the risk of having a stroke. In this disease, there is partial or complete occlusion or narrowing in the terminal part of the carotid artery, seen in arterial imaging.

When these intracerebral vessel changes happen along with other conditions, such as autoimmune diseases, vasculitis, hematological conditions like sickle cell anemia, renal problems like polycystic kidney disease, renal artery narrowing, and any metabolic causes, it is called moyamoya syndrome (MMS).

Our case report describes how we diagnosed and managed a 26-year-old male from Nigeria with MMS.

## Introduction

Moyamoya disease is a rare cerebrovascular disorder that may present with ischemic stroke, hemorrhagic stroke, or TIA. In high-prevalence areas, it is an important cause of stroke in childhood and young adults. Moyamoya disease was first described in Japan and has a higher reported prevalence in East Asian populations, though it is now increasingly recognized worldwide [1]. The disease shows a female predominance (approximately 2:1) and demonstrates a bimodal age distribution, with peaks in the second and fourth decades of life [2].

Sickle cell disease (SCD) is an inherited hemoglobinopathy caused by a point mutation (Glu6Val) in the β-globin gene of chromosome 11, resulting in the production of abnormal hemoglobin S (HbS) [3]. The disorder includes several genotypes, the most common being HbSS (homozygosity for HbS). Other genotypes include hemoglobin SC (HbSC; heterozygosity for HbS with HbC variant) or β-thalassemia (HbSβ thalassemia). 

The greatest prevalence of SCD is seen in people of African and Afro-Caribbean origin, where carrier (sickle cell trait) rates are significant in large parts of sub-Saharan Africa. Sickling of red blood cells can be triggered by various factors that promote deoxygenation or increased metabolic demand, including infection, dehydration, fever, hypoxia, or acidosis. These episodes may present as acute emergencies, such as vaso-occlusive crises [4], and can be life-threatening if not promptly managed.

Under conditions of deoxygenation, HbS polymerizes, leading to deformed erythrocytes resulting in the characteristic sickle shape. The erythrocytes become rigid and obstruct the microvascular flow, leading to ischemia and severe pain, resulting in a vaso-occlusive crisis. 

HbSC disease is a variant of SCD resulting from the co-inheritance of one β-globin gene encoding HbS and one β-globin gene encoding hemoglobin C (HbC), leading to a distinct hematological and clinical phenotype. It is generally regarded as a milder form of SCD compared to homozygous sickle cell anemia (HbSS). However, it can sometimes lead to complications. HbSC disease patients can experience hemolytic anemia, vaso-occlusive crisis, avascular necrosis, and increased risk of stroke. Chronic hemolysis, microvascular occlusion, and hyperviscosity contribute to vascular injury that can lead to moyamoya syndrome (MMS).

The relationship between sickle cell disorders and MMS has been recognized. The condition is most commonly associated with pediatric HbSS, but it can also be seen in adult HbSS as well as HbSC disease. Diagnosis of HbSC disease is made with hemoglobin electrophoresis or HPLC, with supportive evidence from a complete blood count and peripheral smear. 

Treatment for MMS secondary to HbSC disease is directed at preventing stroke and optimizing cerebral perfusion. Medical management includes antiplatelet therapy, hydration, and avoidance of sickling triggers. Chronic transfusion therapy may be used to lower the HbS percentage and prevent further vascular injury. Surgical revascularization procedures may be considered in progressive cases to improve cerebral blood flow.

## Case presentation

A 26-year-old male of Nigerian origin was found confused and disoriented, displaying expressive dysphasia and an unsteady gait. Upon arrival at the emergency department, he was hemodynamically stable with a Glasgow Coma Scale score of 14/15 and normal vital signs. Neurological examination revealed marked expressive dysphasia, word-finding difficulty, and unsteady gait, but no motor or visual deficits were noted. On further examination, there was no clinical evidence of intoxication, infection, or trauma. The patient had no history of chronic illness, allergies, tobacco use, alcohol intake, or illicit substance use. No significant family and social history was identified.

Investigations

Initial biochemistry results on presentation to the emergency department are summarized in Table 1.

**Table 1:** Initial laboratory investigations

Parameters	Patient values	Reference range
Hemoglobin	150 g/L	135-170 g/L
WCC	11.4 × 10^9^/L	3.5-11.0 × 10^9^/L
Platelet	194 x 10^9^/L	140-400 × 10^9^/L
Hematocrit	0.428	0.40-0.51
MCV	80.6 fL	80.0-99.0 fL
MCH	28.2 pg	27.5- 32.5 pg
RBC count	5.31 x 10^12^/L	4.25-6.00 x 10^12^/L
Red cell distribution width	16%	11.6-13.9%
Mean platelet volume	11.2 fL	7.1-10.7 fL
Neutrophil count	9.50 x 10^9^/L	1.70-8.00 × 10^9^/L
Prothrombin time	12.4 s	9.7-12.3 s
INR	1.2	0.9-1.2
aPTT	27.4 s	21-29.0 s
Na	139 mmol/L	133-146 mmol/L
K	3.7 mmol/L	3.5-5.3 mmol/L
Urea	9.1 mmol/L	2.4-7.8 mmol/L
Creatinine	100 umol/L	48-128 umol/L
HbA1C	28 mmol/mol	18-41 mmol/mol

**Figure 1:**
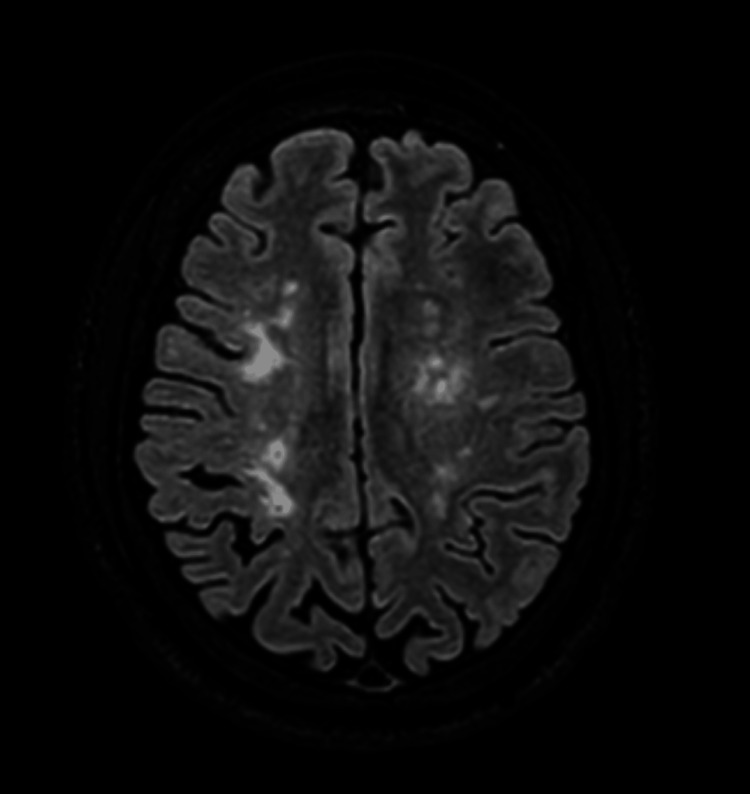
Axial FLAIR image demonstrates parenchymal atrophic changes disproportionate to the patient’s age, along with multiple hyperintense foci consistent with chronic small vessel disease and underlying vascular risk factors

**Figure 2:**
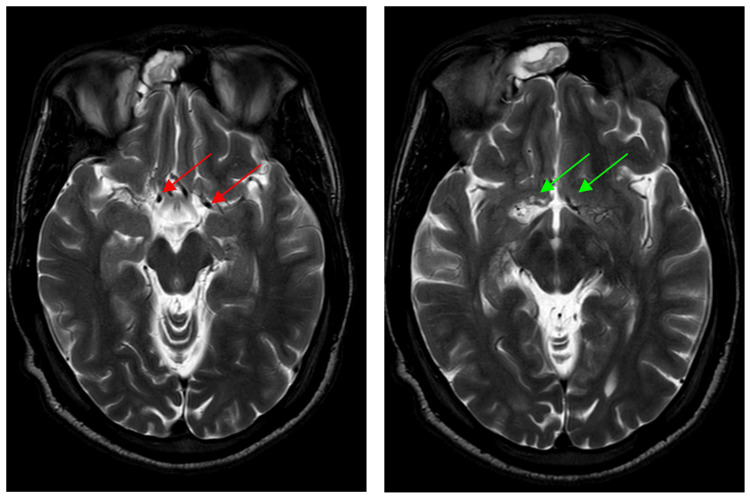
Axial T2 images revealed an unusually small caliber of intracranial vessels, especially along bilateral supraclinoid internal carotid arteries (green arrows) and middle cerebral arteries (red arrows)

**Figure 3:**
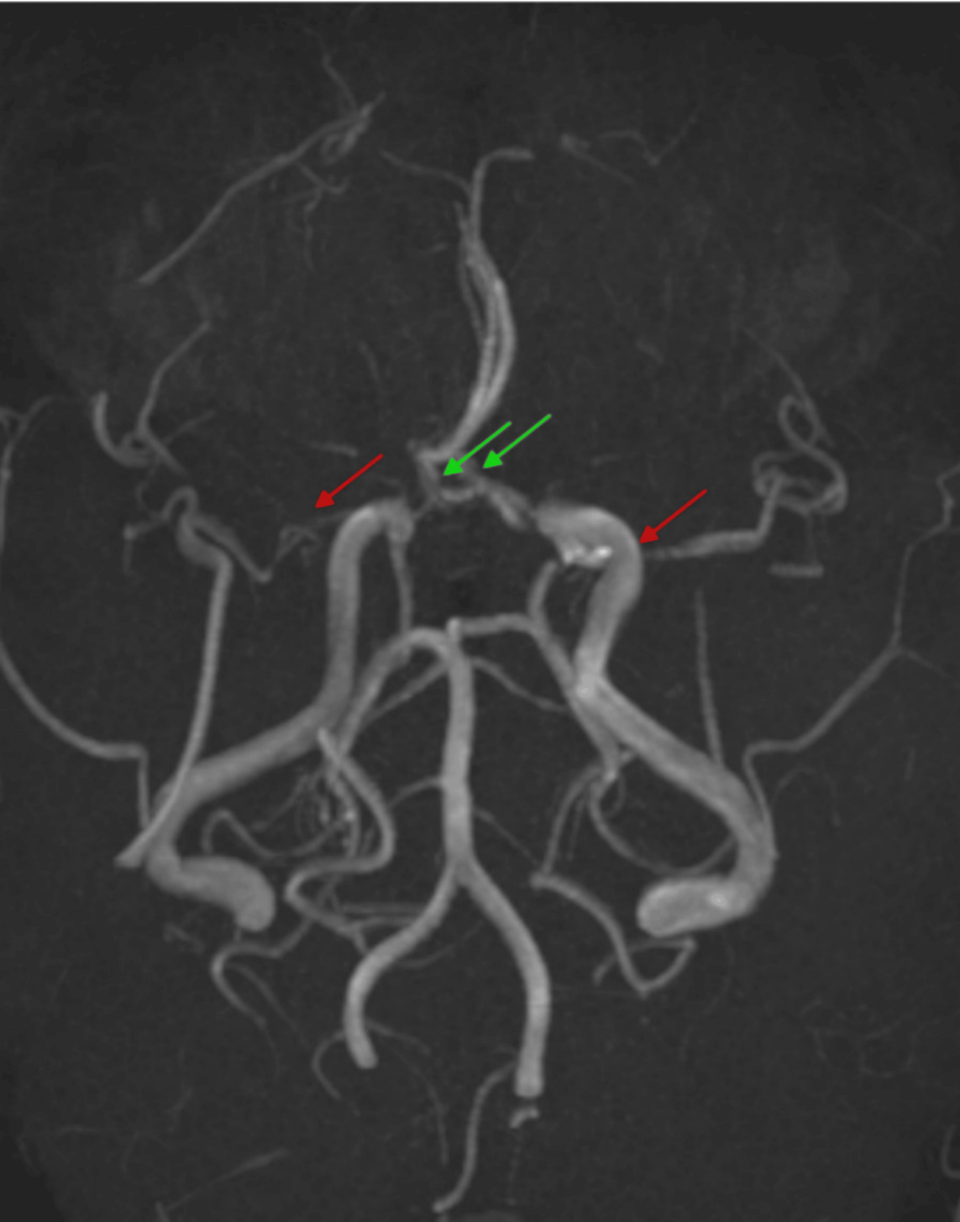
Axial MIP image of time-of-flight magnetic resonance angiography (TOF-MRA) The above image demonstrates near-complete stenosis of the bilateral M1 segments of the middle cerebral arteries (red arrows). The M2 segments and distal branches are visualized but appear attenuated. Significant stenosis is also noted in the bilateral ACA A1 segments (green arrows) just distal to their origin; however, distal flow-related signal within the A2 segments is preserved and within normal limits.

CT imaging of the head demonstrated multiple white matter hypodensities (Figure 1). MRI showed periventricular and subcortical FLAIR hyperintensities with diffusion restriction, initially suggestive of demyelination or small vessel ischemia (Figure 2). Subsequent MRA identified bilateral supraclinoid ICA narrowing and significant stenoses in the MCA, ACA, and left PCoM arteries, consistent with moyamoya phenomenon (Suzuki stage III) (Figure 3).

Hematology findings

A peripheral blood film revealed frequent target cells and occasional sickle cells. The sickle solubility test was positive. Hemoglobin electrophoresis confirmed HbSC disease, with a pre-exchange HbS of approximately 46% and a post-exchange level of 16.1% (Table 2).

**Table 2:** Hematological findings

Parameters	Patient values	Reference range
Protein S (free)	41.7 IU/dL	73-131 IU/dL
Protein C	67 IU/dL	70-140 IU/dL
Anti-thrombin III	74 IU/dL	79-112 IU/dL
Prothrombin 20210 gene screen	Prothrombin gene variant G20210A not detected	Variant absent (normal)
Factor V Leiden genotype	Factor V Leiden mutation not detected	Mutation absent (normal)
Hemoglobin F level	0.7%	0.1-1.0%
Hemoglobin S level	45.9%	0%

Diagnosis

The final diagnosis was an acute bilateral ischemic stroke secondary to sickle cell crisis in the context of previously undiagnosed HbSC disease and sickle cell vasculopathy presenting as moyamoya phenomenon.

Management

The patient was treated with aspirin 300 mg as per the Stroke team management and received an urgent red cell exchange transfusion. He was subsequently transferred to the stroke unit for further multidisciplinary care. Neurovascular MDT referral was made, and inputs from hematology, radiology, and neurosurgery were obtained.

Progress

Significant clinical improvement was noted. He regained mobility, was able to eat and drink independently, and became more oriented. 

Outcome and follow-up plan

The patient was medically optimized and remained clinically stable. The next red cell exchange transfusion was scheduled in four to six weeks. A comprehensive outpatient follow-up plan involving hematology, stroke rehabilitation, and further neurovascular evaluation was initiated.

## Discussion

Stroke is a well-documented complication of SCD, typically affecting children with the homozygous HbSS genotype. By the age of 45, approximately 24% of individuals with SCD will have experienced a stroke. The majority of these events occur during childhood [5]. In contrast, HbSC disease is historically considered a milder variant, with fewer vaso-occlusive crises and a lower stroke incidence. However, this perception is evolving as evidence accumulates that HbSC can also result in significant end-organ damage, including cerebrovascular complications [6,7].

Our case of a 26-year-old male with no prior diagnosis of SCD presenting with stroke as the initial manifestation of HbSC represents a rare and diagnostically challenging scenario. In regions with comprehensive newborn screening, such a delayed diagnosis is unusual. However, in patients born outside such systems, like our patient from Nigeria, HbSC may remain clinically silent until adulthood [8]. This delay in diagnosis often results in missed opportunities for early intervention, family screening, and primary prevention.

MMS, characterized by progressive stenosis of intracranial arteries with compensatory collateral formation, is a known but uncommon complication of SCD. It is classically described in pediatric HbSS populations following recurrent strokes [9]. Cases in adults, particularly with the HbSC genotype, are exceptionally rare.

Our patient is therefore unusual in multiple respects: adult age, absence of prior diagnosis, and HbSC genotype. His presentation with confusion and expressive dysphasia, followed by MRI and MRA showing white matter changes and multivessel intracranial stenosis, confirms the diagnosis of MMS. The blood film findings of sickle and target cells, alongside hemoglobin electrophoresis confirming HbSC, provided the final diagnostic link between the neurological event and the underlying hematological condition.

While most moyamoya cases in SCD are linked to chronic endothelial injury from recurrent sickling, the pathogenesis in HbSC remains less clear. It is hypothesized that chronic low-grade hemolysis and intermittent sickling in HbSC can still lead to progressive vascular damage over time, culminating in moyamoya changes. This theory is supported by isolated reports of other rare haemoglobinopathies, including HbC disease, leading to similar vasculopathy [10].

Imaging findings in our case mirrored those seen in pediatric SCD-moyamoya: bilateral ICA narrowing, extensive collateral formation, and white matter infarction. However, unlike children with recurrent strokes, our patient had no prior neurological symptoms, making this an isolated but severe first presentation. This supports the need for early vascular imaging in young adults with unexplained strokes, particularly those from populations with a higher prevalence of SCD or with suggestive hematological findings.

Management of SCD-related MMS typically involves acute red cell exchange transfusion, followed by chronic transfusion programs to maintain HbS levels below 30% [11]. Our patient improved neurologically following red cell exchange, aligning with previous reports where prompt intervention limited neurological deficits. Long-term management may include neurosurgical evaluation for revascularization procedures, which have shown promising results in reducing stroke recurrence in pediatric SCD-moyamoya cases. Although less frequently performed in adults, case reports support their benefit in select patients [12].

## Conclusions

This case reinforces the importance of recognizing that HbSC is not universally benign. Clinicians should be aware of its potential to cause severe complications, including stroke and MMS. Routine hemoglobin electrophoresis should be considered in young adults with cryptogenic stroke, particularly in those from populations with a higher prevalence of SCD. Early diagnosis allows for timely intervention, appropriate counseling, and family screening.

In summary, this case contributes a rare but important addition to the literature on SCD-associated moyamoya. It demonstrates that stroke can be the first sign of undiagnosed HbSC in adulthood and that such cases, while uncommon, require high clinical suspicion and multidisciplinary management. Early identification and treatment may prevent further neurological injury and improve long-term outcomes.
